# Comparative Analysis of Human Mesenchymal Stem Cells from Umbilical Cord, Dental Pulp, and Menstrual Blood as Sources for Cell Therapy

**DOI:** 10.1155/2016/3516574

**Published:** 2016-01-10

**Authors:** Huaijuan Ren, Yunxia Sang, Fengli Zhang, Zhaoqing Liu, Nianmin Qi, Yantian Chen

**Affiliations:** ^1^Cell Culture and Bioprocess Engineering Lab, School of Pharmacy, Shanghai Jiao Tong University, Dongchuan Road 800, Minhang District, Shanghai 200240, China; ^2^Asia Stem Cell BK, Limited, Shanghai 201203, China

## Abstract

Although mesenchymal stem cells (MSCs) based therapy has been considered as a promising tool for tissue repair and regeneration, the optimal cell source remains unknown. Umbilical cord (UC), dental pulp (DP), and menstrual blood (MB) are easily accessible sources, which make them attractive candidates for MSCs. The goal of this study was to compare the biological characteristics, including morphology, proliferation, antiapoptosis, multilineage differentiation capacity, and immunophenotype of UC-, DP-, and MB-MSCs in order to provide a theoretical basis for clinical selection and application of these cells. As a result, all UC-, DP-, and MB-MSCs have self-renewal capacity and multipotentiality. However, the UC-MSCs seemed to have higher cell proliferation ability, while DP-MSCs may have significant advantages for osteogenic differentiation, lower cell apoptosis, and senescence. These differences may be associated with the different expression level of cytokines, including vascular endothelial growth factor, fibroblast growth factor, keratinocyte growth factor, and hepatocyte growth factor in each of the MSCs. Comprehensively, our results suggest DP-MSCs may be a desired source for clinical applications of cell therapy.

## 1. Introduction

Accumulating evidence has shown that mesenchymal stem cells (MSCs) are an attractive source for tissue engineering and regenerative medicine because of its self-renewal and multilineage differentiation potentials [1, 2]. Although bone marrow and adipose tissues are the main sources for the scientific study and clinic therapy, several of their shortcomings, including decreased proliferation, differentiation potential along with age [3, 4], and the invasive procedure for sample collection, limit their extensive applicability. Therefore, it is of importance to find alternative sources of MSCs to overcome the above key limitations.

Recently, umbilical cord (UC), dental pulp (DP), and menstrual blood (MB) mesenchymal stem cells have gained much attention because of their convenient harvesting procedures, excellent proliferation and differentiation abilities, less susceptibility to bacterial and viral contamination, and no ethical restrictions. Previous studies have reported the therapeutic potential of these MSCs using various models, such as neurodegenerative disorders [5, 6], rheumatoid arthritis [7], hind limb ischemia [8], and diabetes [9], but no direct comparative studies of those three sources of MSCs have been made so far.

The goal of this study was to compare the biological characteristics, including morphology, proliferation, antiapoptosis, multilineage differentiation capacity, and immunophenotype of UC-, DP-, and MB-MSCs in order to select suitable sources of MSCs for future clinical application.

## 2. Materials and Methods

### 2.1. Isolation and Culture of UC-, DP-, and MB-MSCs

This study was approved by Ethics Committee of School of Pharmacy, Shanghai Jiao Tong University, and used protocols of Shanghai Kun'ai Biological Technology Co., LTD. All the donors or their guardians have provided written informed consent.

*UC-MSCs*. Umbilical cords tissues were collected from healthy mothers ranging from 21 to 35 years old after their full-term delivery (cesarean section) and immediately washed with phosphate-buffered saline (PBS) for several times to remove red blood cells. After careful removal of these blood vessels, Wharton's jelly tissues were chopped into small pieces of 0.5–1.5 mm^3^ and plated on the 55 mm^2^ dishes (Corning Incorporated, Corning, NY, USA) for 2 h, followed by being covered with *α*-MEN medium (Gibco, Grand Island, NY, USA) containing 15% fetal bovine serum (FBS, Hyclone, Logan, UT, USA). The medium was changed every three days.

*MB-MSCs*. Menstrual blood was obtained from adult woman aged from 25 to 40 years without vaginal discharge or infection using sterile Diva Cup on the second day of menstrual cycle. The collected menstrual blood (5 mL) was transferred into the tube containing equal volume PBS, 0.2 mL amphotericin B (Invitrogen, Carlsbad, CA, USA), 0.2 mL streptomycin (Invitrogen, Carlsbad, CA, USA), and 0.1 mL EDTA-Na_2_ (Invitrogen, Carlsbad, CA, USA) and mixed at 4°C within 24 h. The blood mononuclear cells were separated twice by density gradient centrifugation at 1500 r/min for 10 min followed by discarding the supernatant and adding another fresh medium containing 15% FBS for cell culture.

*DP-MSCs*. Dental pulp tissues were obtained from shed primary teeth of children aged between 8 and 15 years. The tissues were minced and digested in 10 times the volume of 0.3% collagenase I type (Sigma, St. Louis, MO, USA) at 37°C for 1 h. Subsequently, the *α*-MEM containing 10% FBS was added to terminate digestion, followed by centrifugation at 1000 r/min for 5 min and another addition of 5 times the volume of *α*-MEM containing 15% FBS which was then transferred to 6-well plates and cultured at 37°C, 5% CO_2_ incubation. The medium was replaced every three days.

### 2.2. Immunophenotyping of UC-, DP-, and MB-MSCs

UC-, DP-, and MB-MSCs at passage 2 (P2), passage 6 (P6), and passage 10 (P10) were digested with TrypLE (Invitrogen, Carlsbad, CA, USA), washed twice with PBS, and then suspended in PBS supplemented with 2% FBS at a concentration of 5 × 10^5^ cells/mL. Fluorescein phycoerythrin (PE) conjugated monoclonal antibodies that recognize CD14, CD34, CD45, CD29, CD44, and CD90 (BD Biosciences, San Jose, CA, USA) were added to the cell suspension and incubated in the dark at 4°C. PE conjugated rabbit IgG isotype antibodies (BD Biosciences, San Jose, CA, USA) were used as negative controls. After 30 min incubation, MSCs immunotypes were determined by a FACSAria flow cytometry (BD Bioscience, San Jose, CA, USA) and analyzed using the FlowJo software (Tree Star, Ashland, OR, USA). The percentage of expressed cell surface antigen was calculated for 10,000 gated-cell events.

### 2.3. Multidifferentiation Ability of UC-, DP-, and MB-MSCs

For osteogenic differentiation, the UC-, DP-, and MB-MSCs at P2, P6, and P10 were digested, adjusted to the concentration of 2.5 × 10^4^ cells/mL, and seeded in 12-well plates with *α*-MEM supplemented with 10% FBS. When 80% confluence was reached, the osteogenic induction medium was replaced consisting of H-DMEM supplemented with 10% FBS, 100 nM dexamethasone (DEX, Sigma, St. Louis, MO, USA), 10 mM *β*-glycerol phosphate (Sigma, St. Louis, MO, USA), and 200 *μ*M ascorbic acid (Sigma, St. Louis, MO, USA). The control group was cultured with H-DMEM supplemented with 10% FBS. The medium was renewed every 3 days for 3 weeks. Then, osteogenic differentiation was assayed by Alizarin red (Chroma-Schmidt GmbH, Stuttgart, Germany) staining for calcium deposition and observed by an inverted microscope IX51 (Olympus, Tokyo, Japan). The intensity of the Alizarin red stain was quantified by an enzyme immunoassay analyzer (Thermo, Waltham, MA, USA) at 562 nm after solubilizing in 10% cetylpyridinium chloride (CPC, Sigma, St. Louis, MO, USA) for 10 min at the room temperature.

For adipogenic differentiation, UC-, DP-, and MB-MSCs at P2, P6, and P10 in a concentration of 5 × 10^4^ cells/mL were plated in 12-well plates until 70% confluency. The adipogenic induction medium that consisted of H-DMEM supplemented with 10% FBS, 0.5 mM 3-isobutyl-1-methylxanthine (Sigma, St. Louis, MO, USA), 0.5 mM IBMX, 10 *μ*M insulin (Sigma, St. Louis, MO, USA), and 200 *μ*M indomethacin (Sigma, St. Louis, MO, USA) was then switched and incubated for another 3 weeks. The control group was cultured with H-DMEM supplemented with 10% FBS. The medium was renewed every 3 days. At the end of incubation, adipogenic differentiation was assayed by Oil Red O (Sigma, St. Louis, MO, USA) staining for lipid droplets and observed by an inverted microscope IX51 (Olympus, Tokyo, Japan). Stained cells were quantified by an enzyme immunoassay analyzer (Thermo, Waltham, MA, USA) at 510 nm after eluting with 100% isopropyl alcohol for 5 min at the room temperature.

### 2.4. Proliferation Assay of UC-, DP-, and MB-MSCs

In order to compare the growth kinetics of UC-, DP-, and MB-MSCs, the cells with 80% confluence at different passages (P2, P6, and P10) were digested, adjusted to the concentration of 1 × 10^4^ cells/mL, and seeded on the 24-well plates. At 24 h later, 200 *μ*L/well Alamar Blue (Catalog #DAL1100; Life Technologies, Carlsbad, CA, USA) reagent, a nontoxic metabolic indicator of cell viability [10], was added to each well and plates were incubated for an additional 3 h at 37°C. The plate absorbance was measured using an enzyme immunoassay analyzer (Thermo, Waltham, MA, USA) at 570 nm.

In addition, some wells were trypsinized and the cell number was directly determined on an automatic cell counter (Countstar, Life Technology, Carlsbad, CA, USA) to determine the viability. The population-doubling time (PDT) was calculated for either of the studied MSCs at each passage according to the following equation:

(1) $$\begin{array}{c} PDT=t\times \frac{\mathrm{lg}2}{\mathrm{lg}\left(N_{t}/N_{0}\right)}, \end{array}$$

where *t* indicates the culture time and *N*_*t*_ and *N*_0_ are the numbers of harvesting and initiating cells, respectively.

Furthermore, the metabolic state of MSCs is an important indicator for evaluation of cell viability [11], and the concentrations of glucose and lactate were also monitored at each passage using an SBA-40E biosensor analyzer equipped with glucose and lactate oxidase electrode (Biology Institute of Shandong Academy of Sciences, Shandong, China). The growth curves were drawn after 8 successive days of continuous detection.

### 2.5. Senescence of UC-, DP-, and MB-MSCs

A *β*-galactosidase staining kit (Catalog #GMS10012.1; GENMED, Arlington, MA, USA) was used to detect the senescence in MSCs at P2, P6, and P10 according to the manufacturer's instructions. Senescence was observed under an inverted microscope IX51 (Olympus, Tokyo, Japan) and images were captured with a digital camera (QImaging Go-3; QImaging, Surrey, British Columbia, Canada) using QCapture Pro.6.0 software (Media Cybernetics, Silver Spring, Maryland, USA). Images were semiqualitatively analyzed with the Image Pro Plus 6.0 (IPP6) software (Media Cybernetics, Silver Spring, Maryland, USA) and presented as integrated optical density.

### 2.6. Cellular Apoptosis of UC-, DP-, and MB-MSCs

Apoptotic cell death of UC-, DP-, and MB-MSCs at P2, P6, and P10 was measured via flow cytometry using FITC-conjugated Annexin V/PI assay kit (Catalog #CA001; SAB, Pearland, Texas, USA) [12]. Briefly, the cells were digested, adjusted to the concentration of 1 × 10^5^ cells/mL, and then plated on 6-well plates. After reaching 80% confluence, the cells were collected and suspended in 500 *μ*L buffer, followed by addition of 5 *μ*L Annexin V-FITC and incubation in the dark for 20 min at 4°C. Subsequently, another 10 *μ*L propidium iodide was added and incubated for 5 min. The levels of cellular apoptosis were analyzed by flow cytometry (BD Bioscience, San Jose, CA, USA).

### 2.7. Cytokines Expression of UC-, DP-, and MB-MSCs

The secretion of four cytokines including vascular endothelial growth factor (VEGF) (Catalog #41552; SAB, Pearland, Texas, USA), fibroblast growth factor (FGF) (Catalog #41915; SAB, Pearland, Texas, USA), keratinocyte growth factor (KGF) (Catalog #41962; SAB, Pearland, Texas, USA), and hepatocyte growth factor (HGF) (Catalog #41586; SAB, Pearland, Texas, USA) of UC-, DP-, and MB-MSCs at P2, P6, and P10 was measured by commercial enzyme-linked immunosorbent assay kits according to the manufacture's instruments. Results were acquired by measuring absorbance at 450 nm.

### 2.8. Statistical Analysis

All data were expressed as mean ± standard deviation (SD) from a representative experiment performed in triplicate and were analyzed by SPSS 18.0 (SPSS Inc., Chicago, IL, USA). Differences and significance were verified by one-way analysis of variance (ANOVA) followed by the least-square difference (LSD) (homogeneity of variance) or Dunnett's T3 test (heterogeneity of variance) as a post hoc for multiple comparisons test. *p* < 0.05 was considered statistically significant.

## 3. Results

### 3.1. Distinct Morphology of UC-, DP-, and MB-MSCs

**Figure 1:**
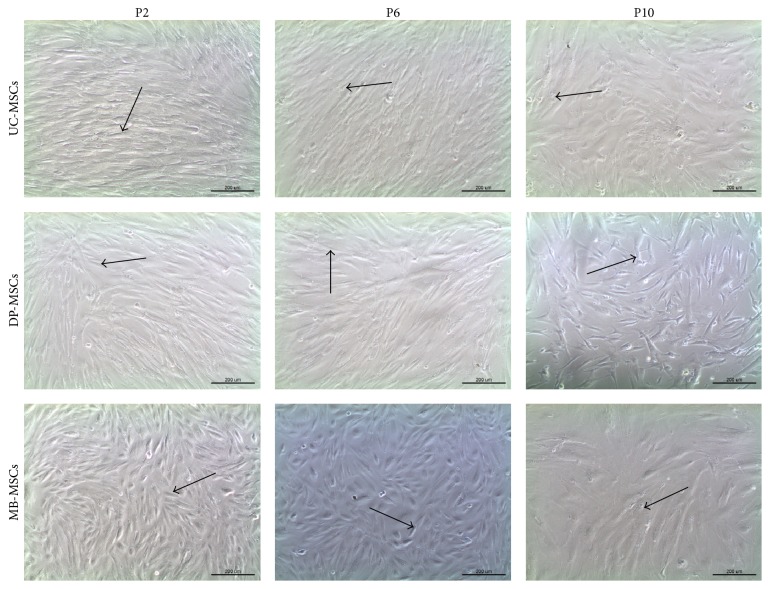
Morphology of UC-, DP-, and MB-MSCs (P2, P6, and P10) (100x). All MSCs exhibited a spindle-shaped morphology at P2 (arrow). However, MB-MSCs gradually became flatted and fragmented at P6 and P10 (arrow); a polygonal shape and cytoplasmic granulations were observed in UC-MSCs at P10 (arrow). A fibroblast-like morphology was maintained in DP-MSCs even at P10 (arrow). UC-MSCs: umbilical cord mesenchymal stem cells; DP-MSCs: dental pulp mesenchymal stem cells; MB-MSCs: menstrual blood mesenchymal stem cells; P: passage.

All MSCs were attached to the surface of culture flask and exhibited a spindle-shaped morphology at early passage (Figure 1). However, along with the cell passaging, flattened cell shape and even debris occurred in MB-MSCs; a polygonal shape and cytoplasmic granulations were displayed in UC-MSCs. DP-MSCs seemed to keep with a good state in fibroblast-like morphology at each passage. These suggested the stem cell morphology can be better maintained in DP-MSCs after subculture.

### 3.2. Expression of Mesenchymal Cell Surface-Specific Markers on UC-, DP-, and MB-MSCs

Flow cytometry analysis revealed that all MSCs were negative for hematopoietic- or endothelial-specific antigens CD14, CD34, and CD45 no matter at early or late passage with the percentage of expressed cell surface antigen <5%. However, they were positive for expression of specific mesenchymal markers CD29, CD44, and CD90 with the percentage of expressed cell surface antigen >95% (Table 1). The notable point was that the expression percentage of CD29, CD44, and CD90 for MB-MSCs at P10 did not reach 95%. These findings indicated that compared with UC- and DP-MSCs the stem cell activity seemed to weaken rapidly in MB-MSCs as the cells repeatedly passaged.

### 3.3. Multidifferentiation Capabilities of UC-, DP-, and MB-MSCs

**Figure 2:**
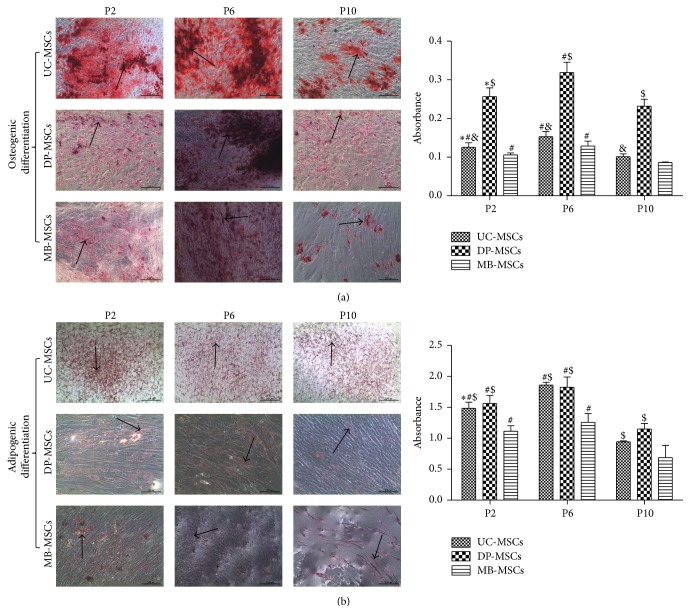
Multidifferentiation of UC-, DP-, and MB-MSCs. (a) Osteogenic differentiation, in which the cells were stained with Alizarin red (100x); (b) adipogenic differentiation in which the cells were stained with Oil Red O (100x). The differentiation capacity was semiquantified by enzyme immunoassay analyzer. Each experiment was performed in triplicate: ^*∗*^compared with P6 of corresponding MSCs; ^#^compared with P10 of corresponding MSCs; ^&^compared with DP-MSCs at corresponding passage; ^$^compared with MB-MSCs at corresponding passage, *p* < 0.05. UC-MSCs: umbilical cord mesenchymal stem cells; DP-MSCs: dental pulp mesenchymal stem cells; MB-MSCs: menstrual blood mesenchymal stem cells; P: passage.

To investigate the differentiation potential, MSCs from three sources were cultured in osteogenic and adipogenic induction medium. Osteogenesis was confirmed by the deposition of red stained calcium, while adipogenesis was determined by the formation of red cytoplasmic lipid droplets. Our results showed that MSCs from three sources can successfully differentiate into osteoblasts (Figure 2(a)) and adipocytes (Figure 2(b)). Further semiquantitative analysis and comparison demonstrated the essentially similar differentiation potential for adipogenesis between DP- and UC-MSCs but significantly better osteogenesis of DP-MSCs compared with UC-MSCs even at the late passage. The MB-MSCs had the significantly lower adipogenic potential than the other two MSCs but the generally similar osteogenic differentiation potential with UC-MSCs at each passage.

### 3.4. Growth Profile of UC-, DP-, and MB-MSCs

**Figure 3:**
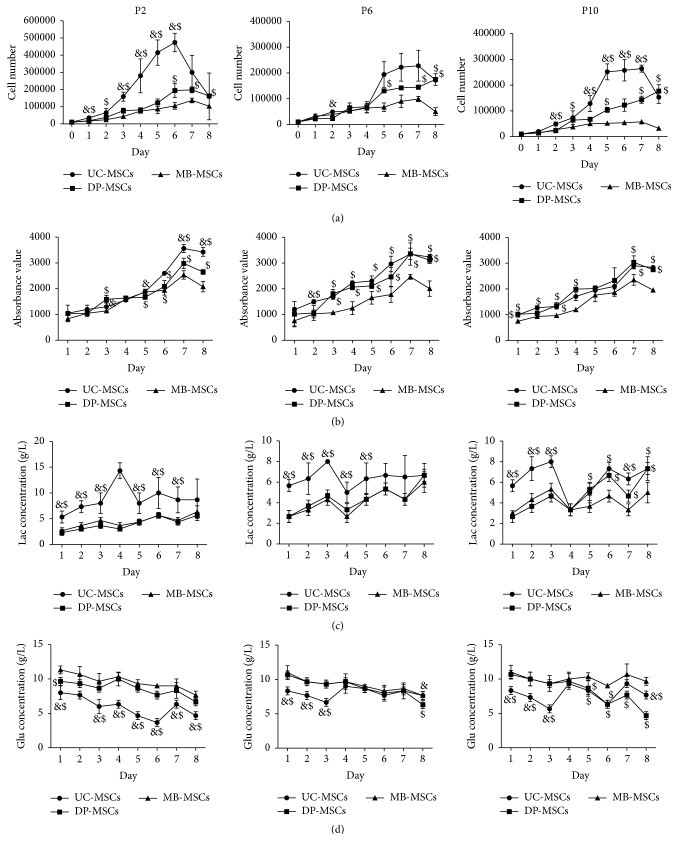
Growth kinetics of UC-, DP-, and MB-MSCs. (a) The cell number determined on an automatic cell counter; (b) cell viability determined by Alamar Blue staining; (c-d) the glucose (Glu) and lactate (Lac) levels monitored using the glucose and lactate biosensors. Each experiment was performed in triplicate: ^&^compared with DP-MSCs at corresponding day; ^$^compared with MB-MSCs at corresponding day, *p* < 0.05. UC-MSCs: umbilical cord mesenchymal stem cells; DP-MSCs: dental pulp mesenchymal stem cells; MB-MSCs: menstrual blood mesenchymal stem cells; P: passage.

**Figure 4:**
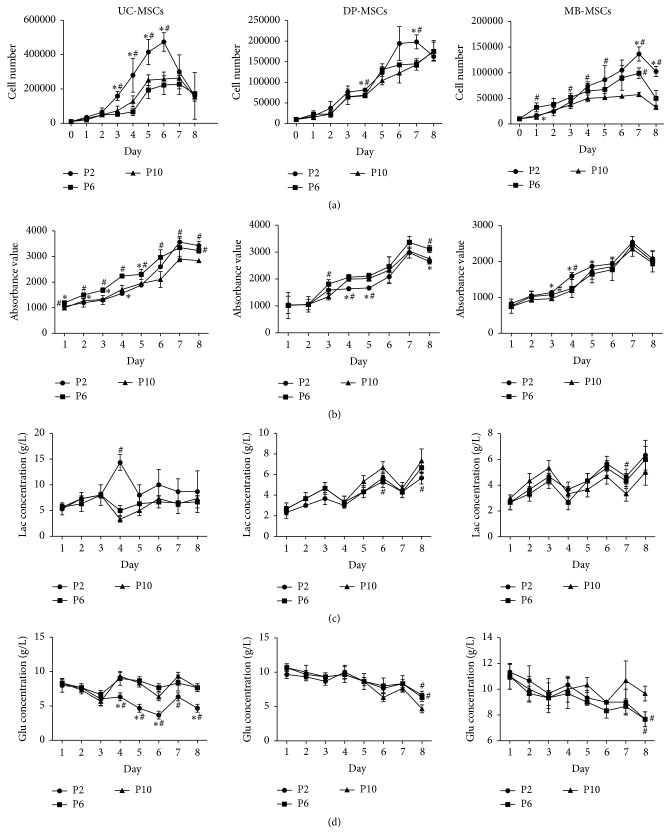
Growth kinetics of UC-, DP-, and MB-MSCs. (a) The cell number determined on an automatic cell counter; (b) cell viability determined by Alamar Blue staining; (c-d) the glucose (Glu) and lactate (Lac) levels monitored using the glucose and lactate biosensors. Each experiment was performed in triplicate: ^*∗*^compared with P6 of corresponding MSCs at corresponding day; ^#^compared with P10 of corresponding MSCs at corresponding day, *p* < 0.05. UC-MSCs: umbilical cord mesenchymal stem cells; DP-MSCs: dental pulp mesenchymal stem cells; MB-MSCs: menstrual blood mesenchymal stem cells; P: passage.

Growth curves of all three MSCs demonstrated that cells proliferated slowly during the first 2 days and then entered the logarithmic growth phase, which continued for approximately 4-5 days, and thereafter reached the cell growth plateau in the days 6-7. UC-MSCs seemed to proliferate significantly faster than DP- and MB-MSCs from the second day (*p* < 0.05, Figure 3(a)). The proliferation difference between DP- and MB-MSCs was only observed in few days, mainly in the logarithmic growth phase. Further, population-doubling time and rate were calculated according to the cell number. As a result, the population-doubling time of the UC-MSCs was found to be significantly less than that of the DP- and MB-MSCs, otherwise for the population-doubling rate at each passage (Table 2). Therefore, UC-MSCs are the most proliferative, followed by DP-MSCs and then MB-MSCs. This trend was further demonstrated by the cell viability analysis stained by Alamar Blue (Figure 3(b)) and metabolic state evaluated by the higher glucose consumption and lactate production (Figures 3(c) and 3(d)). Interestingly, there were little differences in the results of cell number, cell viability, and glucose/lactate levels of each MSC among three different passages (Figure 4), demonstrating that the high expansion capacity still can be maintained at P10.

### 3.5. Senescent Profile of UC-, DP-, and MB-MSCs

**Figure 5:**
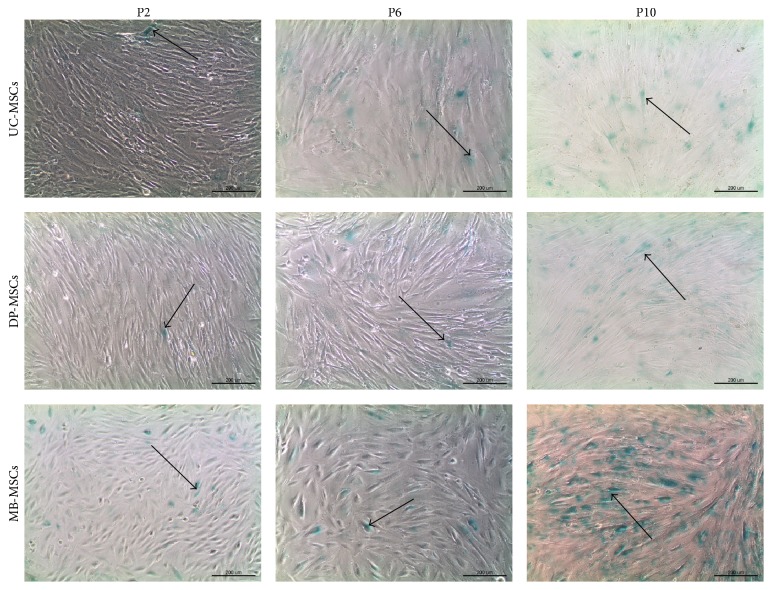
Senescence of UC-, DP-, and MB-MSCs. Senescence was assayed by SA-*β*-gal activity with positive green staining (100x, arrow). UC-MSCs: umbilical cord mesenchymal stem cells; DP-MSCs: dental pulp mesenchymal stem cells; MB-MSCs: menstrual blood mesenchymal stem cells; P: passage.

To determine whether the cellular senescence feature was similar in UC-, DP-, and MB-MSCs, senescence-associated *β*-galactosidase staining was performed. As a result, *β*-galactosidase staining revealedthe least cell senescence occurred in the secondary passage of UC-, DP-, and MB-MSCs. The cell senescence in DP-MSCs was significantly slower than that of the UC- and MB-MSCs at the second and tenth passage, but no significant difference was present between DP- and UC-MSCs at the sixth passage. The MB-MSCs exhibited the most senescent cells compared with the other two MSCs at each passage (Figure 5, Table 3, *p* < 0.05).

### 3.6. Apoptotic Profile of UC-, DP-, and MB-MSCs

**Figure 6:**
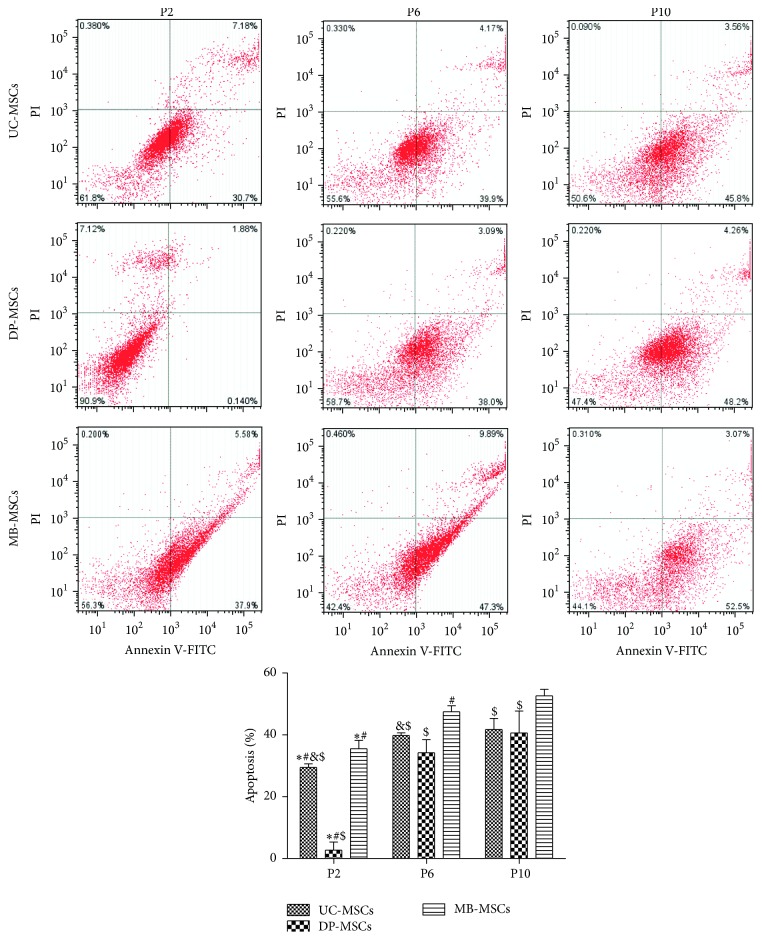
Apoptosis of UC-, DP-, and MB-MSCs. Apoptosis was detected by Annexin V-FITC/PI apoptosis kit and flow cytometry. Each experiment was performed in triplicate: ^*∗*^compared with DP-MSCs at corresponding passage; ^#^compared with MB-MSCs at corresponding passage; ^&^compared with P6 of corresponding MSCs; ^$^compared with P10 of corresponding MSCs, *p* < 0.05. UC-MSCs: umbilical cord mesenchymal stem cells; DP-MSCs: dental pulp mesenchymal stem cells; MB-MSCs: menstrual blood mesenchymal stem cells; P: passage.

In line with the senescence feature, flow cytometry showed that the lowest cell apoptosis occurred in the secondary passage of UC-, DP-, and MB-MSCs, and the apoptosis rate of DP-MSCs was approximately 10-fold lower than that of the UC- and MB-MSCs (Figure 6, *p* < 0.05) in this passage. No significant difference in apoptosis rate was observed between DP- and UC-MSCs at the tenth passage. In addition, the apoptosis rate of UC-MSCs was also significantly lower than that of the MB-MSCs at each passage. Therefore, the antiapoptotic capacity was better in DP-MSCs, followed by UC-MSCs and MB-MSCs.

### 3.7. Expression of Cytokines in UC-, DP-, and MB-MSCs

**Figure 7:**
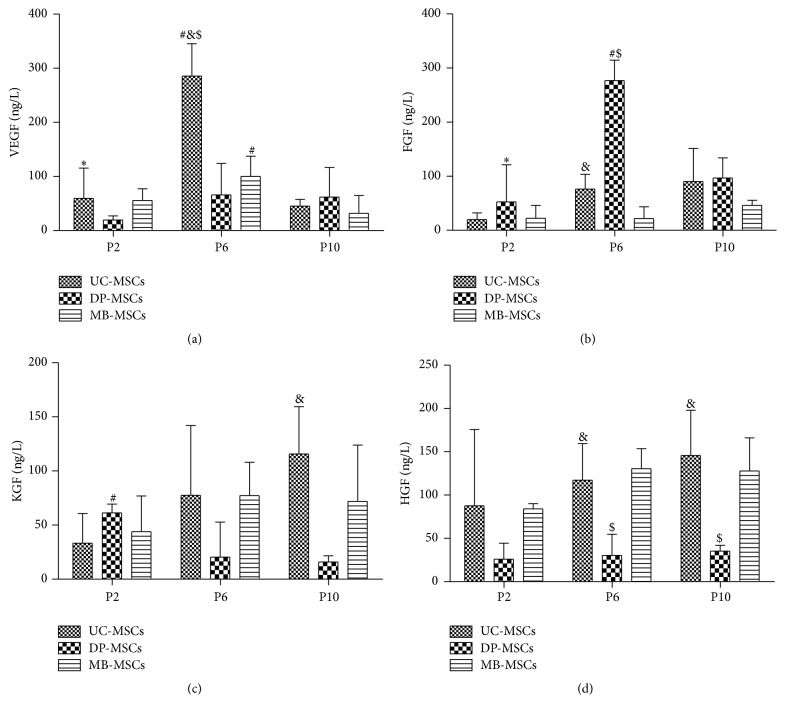
Cytokines of UC-, DP-, and MB-MSCs. (a) VEGF expression; (b) FGF expression; (c) KGF expression; (d) HGF expression. Each experiment was performed in triplicate: ^*∗*^compared with DP-MSCs at corresponding passage; ^#^compared with MB-MSCs at corresponding passage; ^&^compared with P6 of corresponding MSCs; ^$^compared with P10 of corresponding MSCs, *p* < 0.05. UC-MSCs: umbilical cord mesenchymal stem cells; DP-MSCs: dental pulp mesenchymal stem cells; MB-MSCs: menstrual blood mesenchymal stem cells; P: passage; VEGF: vascular endothelial growth factor; FGF: fibroblast growth factor; KGF: keratinocyte growth factor; HGF: hepatocyte growth factor.

Previous studies have shown that the regenerative ability of MSCs is associated with their abilities for secretion of various growth factors, such as VEGF, FGF, KGF, and HGF [13, 14]. Thus, the expressions of VEGF, FGF, KGF, and HGF in UC-, DP-, and MB-MSCs were detected. As shown in Figure 7, the DP-MSCs secreted significant amounts of FGF (276.95 ± 37.55 ng/L) compared with UC-MSCs (76.19 ± 27.23 ng/L) and MB-MSCs (21.75 ± 21.87 ng/L) at the sixth passage. The VEGF and HGF at the sixth (or KGF and HGF at tenth) passage were highly secreted in the UC-MSCs to promote cell proliferation. The concentration of HGF secreted from MB-MSCs was higher than from DP-MSCs at the sixth and tenth passage. No significant difference in four cytokines was observed among three MSCs at the second passage.

## 4. Discussion 

Although MSCs based therapy has been considered to be a promising tool for tissue repair and regeneration, the optimal cell source for clinical application remains unclear. Umbilical cord, dental pulp, and menstrual blood are easily accessible and ethically uncontested sources, which make them attractive candidates for MSCs [15–17]. The goal of this study was to directly compare the therapeutic potentials among the MSCs isolated from these three sources, aiming to provide a theoretical basis for clinical selection and application of these seed cells. This study, to our knowledge, has not been reported.

In large-scale expansion and slow senescence, apoptosis represents the desired characteristics of MSCs for therapeutic purposes. Therefore, the cell proliferation, senescence, and apoptosis in all isolated MSCs were measured in present study. Our results indicated that the proliferation ability of UC-MSCs seemed to be superior to DP-MSCs and MB-MSCs, but the senescence and apoptosis rates were least observed in the DP-MSCs at each passage. More importantly, the stem cells gradually lose their abilities to proliferate but tend to undergo senescence and apoptosis along with the long culture, which was in accordance with previous studies [18–20]. These phenomena may be partially explained by a shift from glycolysis to oxidative phosphorylation energy metabolism pathway [21]. Although more energy can be produced to promote cell proliferation in mitochondrial glucose oxidation (36 moles of adenosine triphosphate (ATP) per mole of glucose) than the glycolysis (only 2 moles of ATP per mole of glucose during the conversion of glucose to pyruvate and then lactate), more reactive oxygen species (ROS) was also released which may ultimately contribute to oxidative stress and cause cell senescence and apoptosis [20, 22]. Thus, scholar believes, like tumors and malignant cells, the maintenance of MSCs predominantly relies on glycolysis instead of mitochondrial glucose oxidation for ATP generation even in the presence of oxygen (aerobic glycolysis or the so-called Warburg phenomenon) [23]. As expected, the UC-MSCs were measured to have higher lactate production than the other two MSCs, especially at P2 in our study.

In addition to energy metabolism, several studies have demonstrated that MSCs secrete growth factors, including VEGF, FGF, KGF, and HGF [24]. It has been reported that MSCs modified by VEGF [25], FGF [26], and HGF [27] gene expression system could significantly enhance cell proliferation* in vitro* and improve the therapeutic effect* in vivo*, but no treatment change occurred when MSCs were transfected with KGF siRNA [28]. However, the excessive proliferation may lead to the nutritional deficiencies. In order to continue survival, the MSCs may autoregulate their own apoptosis and senescence to protect themselves and leave the advantageous population [29, 30]. In line with this hypothesis, our findings revealed growth factors were expressed, but not highly in all MSCs at P2. However, the VEGF, KGF, and HGF were all highly secreted in the UC- and MB-MSCs at P6 and P10, while only FGF was significantly secreted in the DP-MSCs at P6. This reason may lead to relatively less apoptosis and senescence in DP-MSCs compared with others. Additionally, we also can hypothesize that the highly secreted growth factors in the UC- and MB-MSCs at P6 and P10 may be a stress response to maintain cell proliferation, though the effect was not robust [31]. By comprehensive comparison of the cell proliferation, senescence, and apoptosis, the DP-MSCs seemed to be more suitable for clinical application. This conclusion was in accordance with our morphological and surface-specific markers analyses of MSCs which showed the DP-MSCs can maintain the spindle-shaped morphology and positive expression of mesenchymal markers (CD29, CD44, and CD90) even at the P10 passage.

Furthermore, the multidifferentiation ability is another crucial feature for clinical use of MSCs. In this study, the multipotentialities of UC-, DP-, and MB-MSCs were compared by the extent of each cell line's osteogenic and adipogenic differentiation. As a result, the differentiation potential for adipogenesis seemed to be similar among three MSCs, while the differentiation potential for osteogenesis was found to be significantly higher in DP-MSCs than that in UC- and MB-MSCs. These findings are consistent with previous studies [32, 33], which demonstrated that DP-MSCs are capable of quickly differentiating into osteoblasts and, then, able to produce bone tissue, therefore effective for bone engineering. Further studies suggested the high osteogenic differentiation of DP-MSCs may be attributed to the activated NF-*κ*B pathway, which then promoted the expression of osteogenic markers, such as Runx2, collagen type I, and osteocalcin [34, 35]. Furthermore, Kook et al. [36] proved that the secretion of FGF via mitogen-activated protein kinase-mediated signaling pathway may be in favour of osteogenic differentiation of stem cells, which was also preliminarily illustrated by our study, with the highest concentration of FGF and osteogenesis in DP-MSCs at P6 compared with other cells.

## 5. Conclusion 

All human mesenchymal stem cells derived from umbilical cord, dental pulp, and menstrual blood have self-renewal capacity and multipotentiality. Compared with UC- and MB-MSCs, DP-MSCs may have advantages to be used in the treatment of bone injury due to the higher osteogenic differentiation ability and other diseases due to the lower cell apoptosis, senescence, and moderate cell proliferation. However, further comparative experiments are still needed to assess their potentials of neuronal, cardiomyogenic, hepatic, pancreatic, or other types of differentiation and investigate their therapeutic effect* in vivo*.

## Figures and Tables

**Table 1:** Comparison of surface markers of UC-MSCs, DP-MSCs, and MB-MSCs.

		CD29	CD44	CD90	CD14	CD34	CD45
UC-MSCs	P2	99.0 ± 0.3^*∗*#^	98.7 ± 0.6	97.5 ± 0.6^#^	0.7 ± 0.2	0.3 ± 0.3^*∗*^	0.6 ± 0.2^*∗*^
	P6	97.3 ± 1.0	98.1 ± 1.1	97.3 ± 0.6^&#^	0.4 ± 0.1^#$^	1.6 ± 0.5^&$#^	1.3 ± 0.2^&$#^
	P10	97.2 ± 0.8^$^	97.6 ± 0.9^$^	86.2 ± 1.8^&$^	0.9 ± 0.3	0.8 ± 0.3^&^	0.7 ± 0.2^$^

DP-MSCs	P2	99.1 ± 0.2^$*∗*#^	99.7 ± 0.2^$#^	96.9 ± 0.5^*∗*#^	0.4 ± 0.1	0.6 ± 0.2^$^	0.3 ± 0.2^*∗*^
	P6	96.8 ± 0.7^$#^	99.2 ± 0.2^#^	99.6 ± 0.3^$#^	0.5 ± 0.2	0.9 ± 0.3^#^	0.7 ± 0.2
	P10	95.5 ± 0.6^$^	97.1 ± 0.6^$^	98.5 ± 0.5^$^	0.5 ± 0.2^$^	0.3 ± 0.2^$^	0.4 ± 0.2^$^

MB-MSCs	P2	97.4 ± 1.4^#^	98.6 ± 0.7^#^	97.5 ± 0.9^#^	0.6 ± 0.2^#^	0.2 ± 0.1^#^	0.3 ± 0.2^#^
	P6	98.5 ± 0.5^#^	98.4 ± 0.7^#^	97.9 ± 0.7^#^	0.7 ± 0.2^#^	0.4 ± 0.2^#^	0.8 ± 0.3^#^
	P10	75.4 ± 1.4	81.3 ± 0.8	80.6 ± 1.4	1.1 ± 0.2	0.8 ± 0.1	4.5 ± 0.6

^*∗*^Compared with P6 of corresponding MSCs; ^#^compared with P10 of corresponding MSCs; ^&^compared with DP-MSCs at corresponding passage; ^$^compared with MB-MSCs at corresponding passage, *p* < 0.05. More than 95% indicates positive; less than 5% indicates negative. UC-MSCs: umbilical cord mesenchymal stem cells; DP-MSCs: dental pulp mesenchymal stem cells; MB-MSCs: menstrual blood mesenchymal stem cells; P: passage.

**Table 2:** Cell population doubling for UC-MSCs, DP-MSCs, and MB-MSCs.

		UC-MSCs	DP-MSCs	MB-MSCs
P2	PDT (d)	1.020 ± 0.110^*∗*#&$^	1.570 ± 0.046^*∗*#$^	1.873 ± 0.015^#^
	PDR	0.988 ± 0.107^*∗*#&$^	0.637 ± 0.019^*∗*#^	0.534 ± 0.005^#^

P6	PDT (d)	1.553 ± 0.059^&$^	1.947 ± 0.051	1.887 ± 0.025^#^
	PDR	0.644 ± 0.024^&$^	0.517 ± 0.019	0.530 ± 0.007^#^

P10	PDT (d)	1.430 ± 0.085^&$^	2.02 ± 0.040^$^	2.770 ± 0.053
	PDR	0.701 ± 0.042^&$^	0.495 ± 0.010^$^	0.361 ± 0.007

^*∗*^Compared with P6 of corresponding MSCs; ^#^compared with P10 of corresponding MSCs; ^&^compared with DP-MSCs at corresponding passage; ^$^compared with MB-MSCs at corresponding passage, *p* < 0.05. PDT, population-doubling time; PDR, population-doubling rate; UC-MSCs: umbilical cord mesenchymal stem cells; DP-MSCs: dental pulp mesenchymal stem cells; MB-MSCs: menstrual blood mesenchymal stem cells; P: passage.

**Table 3:** Senescence of UC-MSCs, DP-MSCs, and MB-MSCs.

	UC-MSCs	DP-MSCs	MB-MSCs
P2	13962.55 ± 121.30^#&$^	11268.35 ± 368.53^*∗*#$^	18114.90 ± 140.31^*∗*#^
P6	27310.83 ± 11124.41	13501.65 ± 488.90^#$^	51892.55 ± 2070.71^#^
P10	34467.77 ± 672.66^&$^	24289.74 ± 512.88^$^	726805.70 ± 27437.80

The data were presented as integrated optical density. ^*∗*^Compared with P6 of corresponding MSCs; ^#^compared with P10 of corresponding MSCs; ^&^compared with DP-MSCs at corresponding passage; ^$^compared with MB-MSCs at corresponding passage, *p* < 0.05. UC-MSCs: umbilical cord mesenchymal stem cells; DP-MSCs: dental pulp mesenchymal stem cells; MB-MSCs: menstrual blood mesenchymal stem cells; P: passage.
